# Potential of *Aedes aegypti* and *Aedes albopictus* populations in the Central African Republic to transmit enzootic chikungunya virus strains

**DOI:** 10.1186/s13071-017-2101-0

**Published:** 2017-03-27

**Authors:** Carine Ngoagouni, Basile Kamgang, Mirdad Kazanji, Christophe Paupy, Emmanuel Nakouné

**Affiliations:** 1 Institut Pasteur de Bangui, PO Box 923, Bangui, Central African Republic; 2 Research Unit, Liverpool School of Tropical Medicine, Organisation de Coordination pour la lutte contre les Endémies en Afrique Centrale, PO Box 288, Yaoundé, Cameroon; 3 Institut Pasteur de la Guyane, BP 6010, 23 Ave Pasteur, 97306 Cayenne, French Guiana; 4 Laboratoire MIVEGEC, UMR 224-5290 CNRS-IRD-UM, Centre IRD de Montpellier, Montpellier, France

**Keywords:** Chikungunya virus, Enzootic strain, Vector competence, *Aedes aegypti*, *Aedes albopictus*, Central African Republic

## Abstract

**Background:**

Major chikungunya outbreaks have affected several Central African countries during the past decade. The chikungunya virus (CHIKV) was isolated from humans and sylvan mosquitoes in the Central African Republic (CAR) during the 1970 and 1980s but has not been found recently, despite the presence of *Aedes albopictus* since 2010. The risk of a massive chikungunya epidemic is therefore potentially high, as the human populations are immunologically naïve and because of the presence of the mosquito vector. In order to estimate the risk of a large outbreak, we assessed the vector competence of local *Ae. aegypti* and *Ae. albopictus* populations for ancient local strains of CHIKV in CAR. Mosquitoes were orally infected with the virus, and its presence in mosquito saliva was analysed 7 and 14 days post-infection (dpi) by quantitative reverse transcriptase polymerase chain reaction.

**Results:**

The two species had similar infection rates at 7 and 14 days, and the dissemination rate of both vectors was ≥ 80% at 14 dpi. Only females followed up to 14 dpi had CHKV in their saliva.

**Conclusion:**

These results confirm the risk of transmission of enzootic CHIKV by anthropophilic vectors such as *Ae. aegypti* and *Ae. albopictus*.

## Background

The chikungunya virus (CHIKV, Togaviridae, *Alphavirus*) is an *Aedes*-borne virus maintained primarily in African enzootic forest cycles involving monkeys and arboreal mosquitoes [1]. The virus has spilled over secondarily to humans, from whom it was first isolated in Tanzania in 1952 during an epidemic of dengue-like illness [2]. Chikungunya outbreaks were subsequently recorded in rural areas throughout Africa [1], and the virus also spread in Asia during the 1950s and 1960s [3]. Three distinct genotypes of CHIKV have been recognized: Asian, West African and East/Central/South African [4]. In the 2000s, the virus induced major epidemiological changes by extending its geographical range to new continents, causing epidemic urban waves [5]. The Asian lineage spread in the Americas [6] and the South Pacific region [7] and the East/Central/South African lineage to the south-west Indian Ocean, including La Réunion [8] and India [9], and to Europe [10]. In Central Africa, the virus was previously observed only during rural epidemics, but it has resurged, causing major urban outbreaks in Cameroon, Congo and Gabon between 2006 and 2011 [11–13]. In Gabon, the main vector species was *Ae. albopictus* [14], even in remote villages in the heart of the rainforest [15].

The growing epidemic role of *Ae. albopictus* in Central Africa is, however, not restricted to CHIKV: the species was also involved in the transmission of dengue virus [16] and Zika virus [17], suggesting that this vector has strongly affected the epidemiology of a number of arboviruses in the region. This species was introduced in 2000 in Cameroon and spread quickly to most countries of Central Africa [18], where it has outcompeted the native vector *Ae. aegypti* to become dominant in some urban locations in Cameroon, CAR and Gabon [19]. Molecular studies of East/Central/South African viral strains circulating in areas where *Ae. albopictus* was the primary vector have shown a specific change in the amino acid composition of the membrane fusion glycoprotein (change E1-A226V), which was interpreted as an adaptation of the virus for enhanced transmission by *Ae. albopictus* [20]. Genetic comparisons of CHIKV strains circulating recently in Central Africa with strains isolated between 1975 and 1984 in CAR showed significant differences compatible with rapid adaptation to *Ae. albopictus*, as observed previously in other countries in the region [21]. *Aedes albopictus* was reported for the first time in CAR in 2010 [22] and became the most prevalent *Aedes* mosquito species, outcompeting the native *Ae. aegypti* [23].

*Aedes albopictus* has strong ecological plasticity, which allows it to adapt rapidly to a wide range of habitats [18]. For instance, although *Ae. albopictus* originated in Asian forests, it has now adapted to a range of rural and urban human environments. Females of *Ae. albopictus* are opportunistic feeders and can take their blood meal from most groups of vertebrates, from cold- to warm-blooded animals, including reptiles, birds and amphibians, although they display a marked biting preference for mammals, including humans [18]. This broad spectrum of host preferences contributes to the invasion and settlement of *Ae. albopictus* in diverse environments, from natural ones to densely populated urban areas. At the interface between natural and anthropogenic environments, its zoophilic behaviour can transfer enzootic arboviruses from animals to humans. To assess this possibility, we determined the oral susceptibility of *Ae. aegypti* and *Ae. albopictus* from CAR to enzootic CHIKV strain isolated in the country.

## Methods

*Aedes aegypti* and *Ae. albopictus* were sampled in 2014 in Bangui (04°21'N, 18°33'E), the capital of CAR. For each species, larvae and pupae were collected in several peri-domestic containers and reared to adulthood in the insectary of the Institut Pasteur de Bangui under controlled conditions (70–80% humidity, 28 ± 1 °C). Once identified as *Ae. aegypti* or *Ae. albopictus*, adults were pooled according to species in separate cages with free access to a 10% sucrose solution until experimental infection with CHIKV.

The CHIKV strain used for experimental infection of mosquitos was an enzootic strain (ArB10262) isolated in CAR in 1978 from *Ae. africanus* in forested environments in Bozo (5°7'60"N, 10°28'60"E)*.* The virus was stored at the Institut Pasteur de Bangui in a lyophilized form in sealed glass vials at room temperature. This strain is one of 15 CHIKV strains sequenced and analysed recently by Desdouits and collaborators [21]. The virus was suspended in 2 ml of phosphate buffered saline and inoculated into an approximately 80% confluent Vero E6 cell line (two passages) until strong cytopathic effects were seen. Cells and supernatant were recovered from the second passage and stored at -80 °C until experimental mosquito infections.

Infection experiments were performed in the BSL-3 laboratory at the Institut Pasteur de Bangui with 5–10 day-old female mosquitos. About 50–100 females were deprived of sugar solution for 24 h and fed the infectious meal with a Hemotek feeding system (Discovery workshops, Accrinton, UK). The infectious blood meal was composed of a virus suspension diluted (1:3) in washed rabbit erythrocytes. Adenosine triphosphate, a phagostimulant, was added at a final concentration of 5 × 10^-3^ mol/l [24]. An aliquot of the infectious blood meal was used to estimate the blood-meal titre in a plaque assay, which was found to be 10^8^ plaque-forming units per ml. Mosquitoes were allowed to feed for 45 min on a membrane feeder maintained at 37 °C. Fully engorged females were transferred in small cardboard containers and maintained at 28 ± 1 °C and 75% relative humidity with a 10% sugar solution. Surviving females were removed at 7 and 14 days post-infection (dpi) and tested for the presence of CHIKV in saliva and head. In each experiment, control females were fed a non-infectious blood meal.

Saliva was collected 7 and 14 dpi by the forced salivation technique according to the protocol described by Dubrulle and collaborators [25] to estimate dissemination and transmission rates. As most studies show that CHIKV is found early (around 7 dpi), we assessed whether the infection and/or dissemination rates increased or decreased between 7 and 14 dpi. Briefly, wings and legs were removed from each mosquito, and the proboscis was introduced into a micropipette containing 5 μl of fetal bovine serum. After 30–45 min of salivation, fetal bovine serum containing saliva was expelled into 45 μl of Leibovitz L15 medium (Invitrogen), and individual females were dissected to remove the head (as well as the thorax and abdomen) to estimate dissemination and transmission rates. All biological material was stored at -80 °C until processing for quantitative reverse transcriptase polymerase chain reaction (qRT-PCR).

Mosquito tissue was ground in 600 μl of sterile phosphate buffered saline 1×. RNA was extracted with a mini QIAamp RNA Viral kit (Qiagen, Hilden, Germany) according to the manufacturer’s recommendations, eluted in 50 μl elution buffer and stored at -80 °C until analysis. RNA retro-transcription was performed with 25 μl of the RNA template and random hexamer primers in the high-capacity cDNA reverse transcription kit (Applied Biosystems, USA), according to the manufacturers’ instructions. qRT-PCR was conducted in an ABI 7500 Fast Real-time PCR System and the Taqman Universal PCR Master Mix (Applied Biosystems, Foster City, CA, USA). Amplification was performed in a final volume of 25 μl containing 5 μl cDNA, 12.5 μl 2× Master Mix, 1 μl of each primer (10 mmol/l) and 4.5 μl of sterile water. The primers and probe sequences used have been described by Pastorino and collaborators [26], as follows: forward primer: 5′-AAG CTY CGC GTC CTT TAC CAA G-3′, reverse primer: 5′-CCA AAT TGT CCY GGT CTT CCT-3′, and probe 5′ FAM-CCA ATG TCY TCM GCC TGG ACA CCT TT-TAMRA 3′. The thermocycling conditions were: 50 °C for 2 min, 95 °C for 10 min, followed by 45 cycles of 95 °C for 15 s and 60 °C for 10 min.

The three parameters assessed were infection, dissemination and transmission rates. The infection rate was expressed as the percentage of all blood-fed mosquitoes tested that had infected bodies (abdomen). The dissemination rate is the percentage of mosquitoes among all infected mosquitoes that have CHIKV RNA in the head. The transmission rate is the number of mosquitoes with infected saliva divided by the number of mosquitoes with infected head plus thorax. Fisher’s exact test was used to compare infection, dissemination and transmission rates between the two species at 7 and 14 dpi with STATA/IC version 11 (StataCorp, College Station, TX). Differences were considered statistically significant when *P* < 0.05.

## Results and disscussion

We analysed 75 *Ae. aegypti* and 66 *Ae. albopictus* engorged females. For *Ae. aegypti*, 16 of 75 engorged females were analysed at 7 dpi and the rest (*n* = 59) at 14 dpi, and, for *Ae. albopictus*, 22 of 66 were tested at 7 dpi and 44 at 14 dpi. At 7 dpi, the infection rates were comparable (Fisher’s exact test, *P* = 0.32, Table 1), whereas, at 14 dpi, the infection rate was more than twice as high in *Ae. aegypti* (27%) than in *Ae. albopictus* (11%), although the difference was not significant (Fisher’s exact test, *P* = 0.08, Table 1). At 7 dpi, the dissemination rates were 87% in *Ae. aegypti* and 42% in *Ae. albopictus*, but with no statistically significant difference (Fisher’s exact test, *P* = 0.11, Table 1). At 14 dpi, the dissemination rates were similar in the two species, at 87 and 80%. No positive saliva samples were found at 7 dpi in either species, but at 14 dpi CHIKV was detected in the saliva of four *Ae. aegypti* (28%) and three *Ae. albopictus* (75%) mosquitos, with comparable transmission rates (Fisher’s exact test, *P* = 0.24, Table 1). All pools (unexposed) included in the experiment were negative. The low infection rates found in both species may be due to the origin of the CHIKV strain in the sylvan mosquito *Ae. africanus*.

**Table 1:** Infection, dissemination and transmission rates calculated at 7 and 14 days post-infection (dpi) for *Aedes aegypti* and *Ae. albopictus* females orally challenged with CHIKV at a titre of 10^7.3^ plaque-forming units per ml

	Infection rate (%)		Dissemination rate (%)		Transmission rate (%)	
Mosquito species	7 dpi	14 dpi	7 dpi	14 dpi	7 dpi	14 dpi
*Aedes aegypti*	8/16 (50)	16/59 (27)	7/8 (87)	14/16 (87)	0/7 (0)	4/14 (28)
*Aedes albopictus*	7/22 (31)	5/44 (11)	3/7 (42)	4/5 (80)	0/3 (0)	3/4 (75)
*P* ^a^	0.32	0.08	0.11	1.00	–	0.24

^a^Fisher’s exact test (*P* < 0.05 indicates significant difference)

Emerging and re-emerging *Aedes*-borne diseases such as dengue, chikungunya and Zika are major threats in the tropics and also in temperate countries. Global changes have played an important role in the spread of these arboviruses from their original niches to most parts of the world. Yellow fever, which is an arbovirus disease that can be prevented by vaccination, recently reappeared in Angola before spreading to Kenya and to China [27]. As numerous arboviruses were isolated in the past in Central Africa [17, 28], this region is considered an area of active circulation of different arboviruses and consequently represents a source of viruses that are “concealed” in other parts of the world by human displacement. Our findings show that *Ae. aegypti* and *Ae. albopictus* can transmit the CHIKV strain of enzootic origin, as viral particles were detected in saliva at 14 dpi, with 5.4 ± 0.8 log_10_ and 5.1 ± 0.4 log_10_ particles from *Ae. aegypti* and *Ae. albopictus*, respectively.

Since the outbreak of chikungunya in La Réunion due to a viral strain harbouring substitution of an alanine to a valine at position 226 (E1-A226V) of the E1 glycoprotein, which enhanced the transmissibility of CHIKV by *Ae. albopictus* [20, 29], numerous studies have been conducted worldwide on the vector competence of *Ae. aegypti* and *Ae. albopictus* populations [14, 25, 30, 31]. Nevertheless, only a few have been conducted in Cameroon [14] and more recently in Senegal [32], providing information on Africa. In our study, the dissemination rates were higher (≥ 80%) at 14 dpi, and mosquitoes were susceptible to transmitting CHIKV only 14 days after they had taken the infected meal. In a study by Vega-Rua and collaborators [33], the dissemination rates increased to 100% at 6 dpi in *Ae. aegypti* and *Ae. albopictus* infected with two CHIKV strains, one of which was isolated from imported cases (CHIKV 2010–1630) and the second in France (CHIKV 2010–1909); the titre of the blood meal used was 10^7.3^ plaque-forming units per ml. Other studies have shown efficient dissemination of the E1-226 V variant, which was detectable in the salivary glands of *Ae. aegypti* and *Ae. albopictus* from 2 dpi [25]. More recently, Vega-Rua and collaborators [33] showed that around 80% of both species can transmit CHIKV at 6 dpi, but the dissemination rate decreased progressively to day 14. The extrinsic incubation periods observed in the present study are different from that observed by Diagne et al. [32] using local CHIKV strains, which was 5 days for *Ae. aegypti*.

The CHIKV strain used in our study was isolated from a sylvatic vector, *Ae. africanus*. Desdouits and collaborators [21] showed that this strain differs from the East/Central/South African strain responsible for the recent chikungunya outbreaks reported in numerous Central African countries. The ability of these major vectors to transmit arboviruses has not previously been assessed in CAR, although many important viral strains, including CHIKV, are endemic and despite the risk of imminent outbreaks of arboviral infections such as chikungunya in the country [23]. Our finding that *Ae. aegypti* and *Ae. albopictus*, which are the main vectors involved in the transmission of dengue virus and CHIKV in urban environments, can also transmit a local CHIKV strain suggests that both vectors can serve as a bridge between non-human primates and humans and that there is a risk for the emergence of this type of virus, as suggested by Paupy et al. [15]. In addition, if the virus is transferred to a human-dominated environment (city or village), inter-human transmission might occur, leading to outbreaks.

## Conclusion

Both local *Ae. aegypti* and *Ae. albopictus* populations can transmit an enzootic CHIKV. The risk of emergence of arbovirus diseases such as chikungunya should therefore be considered for several reasons, including the abundance of the main anthropogenic vectors (*Ae. aegypti* and *Ae. albopictus*), the movement of populations that are immunologically naïve and the diversity of wild fauna. In order to prevent the emergence of chikungunya in CAR, entomological and virological monitoring must be strengthened.

## Abbreviations

CAR: Central African Republic
CHIKV: Chikungunya virus
dpi: Day post-infection
qRT-PCR: Quantitative reverse transcription polymerase chain reaction
